# Insulin and branched-chain amino acid depletion during mouse preimplantation embryo culture programmes body weight gain and raised blood pressure during early postnatal life

**DOI:** 10.1016/j.bbadis.2017.11.020

**Published:** 2018-02

**Authors:** Miguel A. Velazquez, Bhavwanti Sheth, Stephanie J. Smith, Judith J. Eckert, Clive Osmond, Tom P. Fleming

**Affiliations:** a Biological Sciences, University of Southampton, Southampton General Hospital, Southampton SO16 6YD, UK; b School of Natural and Environmental Sciences, Newcastle University, Newcastle Upon Tyne NE1 7RU, UK; c Human Development and Health, Faculty of Medicine, University of Southampton, Southampton General Hospital, Southampton SO16 6YD, UK; d MRC Lifecourse Epidemiology Unit, University of Southampton, Southampton General Hospital, Southampton SO16 6YD, UK

**Keywords:** Blastocyst, Insulin, Branched-chain amino acids, Birth weight, Systolic blood pressure, DOHaD (developmental origins of health and disease)

## Abstract

Mouse maternal low protein diet exclusively during preimplantation development (Emb-LPD) is sufficient to programme altered growth and cardiovascular dysfunction in offspring. Here, we use an in vitro model comprising preimplantation culture in medium depleted in insulin and branched-chain amino acids (BCAA), two proposed embryo programming inductive factors from Emb-LPD studies, to examine the consequences for blastocyst organisation and, after embryo transfer (ET), postnatal disease origin. Two-cell embryos were cultured to blastocyst stage in defined KSOM medium supplemented with four combinations of insulin and BCAA concentrations. Control medium contained serum insulin and uterine luminal fluid amino acid concentrations (including BCAA) found in control mothers from the maternal diet model (N-insulin + N-bcaa). Experimental medium (three groups) contained 50% reduction in insulin and/or BCAA (L-insulin + N-bcaa, N-insulin + L-bcaa, and L-insulin + N-bcaa). Lineage-specific cell numbers of resultant blastocysts were not affected by treatment. Following ET, a combined depletion of insulin and BCAA during embryo culture induced a non sex-specific increase in birth weight and weight gain during early postnatal life. Furthermore, male offspring displayed relative hypertension and female offspring reduced heart/body weight, both characteristics of Emb-LPD offspring. Combined depletion of metabolites also resulted in a strong positive correlation between body weight and glucose metabolism that was absent in the control group. Our results support the notion that composition of preimplantation culture medium can programme development and associate with disease origin affecting postnatal growth and cardiovascular phenotypes and implicate two important nutritional mediators in the inductive mechanism. Our data also have implications for human assisted reproductive treatment (ART) practice.

## Highlights

• Chronic disease may derive from maternal undernutrition during pregnancy, including the periconceptional period.
• Mouse embryos cultured in medium low in insulin and select amino acids gave rise to offspring with disease symptoms.
• We propose these metabolite deficiencies around conception induce adverse programming of the early embryo leading to increased disease risk in later life.

## 1 Introduction

Undernutrition is a worldwide concern affecting not only countries with developing and emerging economies but also populations in countries with a high human development index [1]. Human epidemiological studies have revealed that undernutrition during the prenatal period can increase the risk of developing non-communicable diseases (NCDs) in adulthood [2], [3]. Indeed, experimental research in animal models has provided strong evidence that prenatal undernutrition can program the occurrence of altered phenotypes (e.g. increased blood pressure) in postnatal life [3]. This unfavourable programming is the basis for the developmental origins of health and disease (DOHaD) hypothesis [2]. Such an adverse programming can be induced at several developmental stages during the prenatal period, including the preimplantation phase of embryo development [4].

In a murine model of protein restriction it was shown that dams fed with a low protein diet (9% casein) exclusively during the preimplantation period (days 0–3.5 of embryonic development; Emb-LPD) produced offspring that displayed altered phenotypes during postnatal life, including increased postnatal growth, high blood pressure, vascular dysfunction and hyperactive behavior [5], [6], [7]. This animal model of undernutrition has also revealed that compensatory mechanisms exist to maintain viable growth of the developing fetus by altering cellular characteristics of the placental lineages. For instance, compared to the control group (18% casein), protein-restricted females (i.e. Emb-LPD) produced blastocysts with a higher number of cells in the trophectoderm (TE) [8], augmented endocytic activity in TE cells [9], and increased spreading capacity during in vitro outgrowth formation [8]. Later in gestation, ectoplacental cones collected at embryonic day 8 (E8.5) from Emb-LPD females and cultured in vitro for 24 h displayed an increased spreading area along with decreased number of secondary trophoblast giant cells [10]. Similarly, in the primitive endoderm lineage and derivative yolk sac placenta, increased endocytic activity is stimulated by maternal protein restriction [5], [9]. These changes are associated with an increased fetal:placental weight ratio due to development of larger fetuses with smaller placentas [10]. These phenotypic alterations seem to be induced at the blastocyst stage, around the time of cell lineage determination, since recipients fed with normal levels of protein receiving protein-restricted embryos through embryo transfer produced conceptuses with increased weight [5].

In the Emb-LPD murine model, decreased levels of insulin in blood and branched-chain amino acids (BCAA) in uterine luminal fluid (ULF) were detected at the time of blastocyst formation and coincided with a reduced blastocyst mTORC1 signal mediated through these metabolites [8]. In vitro experiments have revealed that exposure to insulin and amino acids (AA) during the preimplantation period can affect not only early embryo development [11], [12], [13] but also fetal growth [14], [15], [16]. However, the possible long-term effects of fluctuations of these nutritional mediators during the preimplantation period on postnatal development are currently unknown. This type of research is critical for the elucidation of the mechanisms behind the adverse programming of chronic disease during prenatal undernutrition. Hence, in the present study we test the hypothesis that insulin and/or BCAA depletion during preimplantation embryo development can act as inductive factors of altered phenotypes during postnatal life. Using an in vitro embryo culture (IVEC) and embryo transfer (ET) model we provide evidence that exposure to low levels of insulin and BCAA exclusively during preimplantation embryo development is sufficient to alter body weight gain and blood pressure during early postnatal life in mice.

## 2 Materials and methods

### 2.1 Animals

Outbred MF1 mice under UK Home Office Licence were bred in-house (Biomedical Research Facility, University of Southampton) on a 0700–1900 light cycle. Experimental procedures were conducted using protocols approved by, and in accordance with, the UK Home Office Animal (Scientific Procedures) Act 1986 and local ethics committee at the University of Southampton. All males and females used for embryo production or ET were fed with standard chow and water ad libitum at all times (i.e. mating, pregnancy and lactation).

### 2.2 Embryo collection

Non-superovulated virgin MF1 females (7–8.5 weeks) were mated (1:1) overnight with MF1 males. Presence of copulation plugs was checked the following morning and regarded as a sign of successful mating. Plug-positive females were considered to be on embryonic day 0.5 (E0.5) at midday on the day the vaginal plug was detected. Pregnant females were caged in groups of two to four. Mice were killed by cervical dislocation and oviducts were immediately dissected on E1.5 to collect two-cell embryos. Oviducts were placed in warm (37 °C) saline solution (BR0053G, OXOID, UK) and then transferred to an empty petri dish where they were gently flushed under a stereomicroscope with 0.5 ml of H6 medium supplemented with 4 mg/ml bovine serum albumin (BSA), (A3311, Sigma, UK) [17]. Embryos were then washed with fresh H6-BSA to remove debris.

### 2.3 In vitro embryo culture

**Fig. 1:**
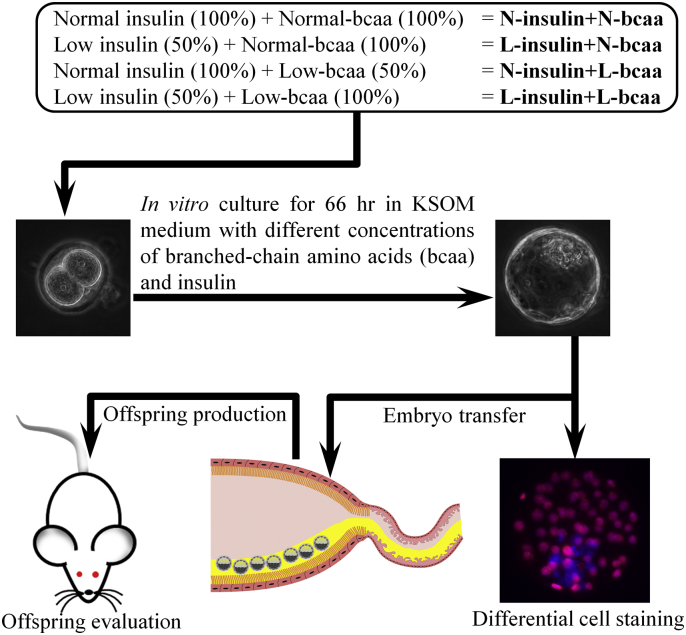
Schematic representation of the murine experimental model used in this study. The effects of branched-chain amino acids (bcaa) and/or insulin depletion (Low, L = 50%) on in vitro blastocyst development and health of the resultant offspring following embryo transfer was studied. Two-cell embryos were in vitro cultured for 66 h until the blastocyst stage. The control medium was termed “normal” (N) and represented 100% of the serum insulin levels and uterine luminal fluid amino acid concentrations (N-Insulin + N-bcaa), including bcaa, found in pregnant (E3.5) mice fed with normal levels of protein (18% casein) taken from [8].

Two-cell embryos were randomly allocated to different concentrations of insulin and BCAA in groups of 11–15 in 30 μl drops and cultured up to the blastocyst stage for 66 h. Microdrops were covered with mineral oil (M8410, Sigma, UK) and incubated under 5% CO_2_ in air at 37 °C. The basic in vitro culture medium was potassium simplex optimized medium (KSOM) where BSA was omitted and ethylenediaminetetraacetic acid (EDTA) at non-toxic concentrations (0.01 mM) was added to allow a chemically defined milieu for embryo culture [18], [19]. The control KSOM medium was supplemented with serum insulin levels and uterine luminal fluid amino acid (AA) concentrations, including BCAA, found in pregnant (E3.5) MF1 mice fed with normal levels of protein (18% casein) [8]. The concentrations of insulin (1 ng/ml) and BCAA (valine = 0.46 mM, isoleucine = 0.21 mM, leucine = 0.32 mM) used in the control medium were termed “normal” (N-insulin + N-bcaa) and represented 100% of the levels found in vivo. The concentrations of amino acids used are shown in Table 1. The control group was compared with three experimental groups where insulin and BCAA were either low (50%) or constant (100%), giving the following combinations: L-insulin + N-bcaa, N-insulin + L-bcaa, and L-insulin + N-bcaa (Fig. 1). Our in vitro model intended to mimic our in vivo model of protein restriction where the Emb-LPD treatment reduced maternal serum insulin by approximately 40% and BCAA concentrations in ULF by approximately 30% at the time of blastocyst formation (E3.5) [8]. To remove H6-BSA medium, embryos were washed three times in their respective culture medium before in vitro culture.

**Table 1:** Amino acid composition of control medium (N-insulin + N-bcaa).

Amino acid	Concentration (mM)
Alanine	3.8
Arginine	0.2
Asparagine	0.1
Aspartic ac id	1.8
Cysteine	0.2
Glutamic acid	4.7
Glutamine	1.4
Glycine	2.7
Histidine	0.1
Isoleucine	0.2
Leucine	0.3
Lysine	0.5
Methionine	0.2
Phenylalanine	0.1
Proline	0.1
Serine	1.0
Taurine	14.7
Threonine	0.7
Tryptophan	0.06
Tyrosine	0.2
Valine	0.5

### 2.4 Differential cell staining in blastocysts

Differential nuclear labelling was carried out in expanded blastocysts (Fig. 1) based on the protocol developed by [20] with some modifications as previously described [21]. Briefly, zona pellucidae were removed with warm (37 °C) acid Tyrode's solution (T1788, Sigma, UK) followed by 15–20 min washing in H6-BSA. Zona-free blastocysts were incubated 10 min in 10% trinitrobenzenesulfonic acid solution (TNBS, P-2297, Sigma, UK) at room temperature. Embryos were then washed three times with H6-PVP, incubated for 10 min in 0.4 mg/ml goat anti-dinitrophenyl (anti-DNP) antibody (D9781, Sigma, UK) in H6-PVP at room temperature, washed again three times with H6-PVP and incubated in 50 μl drops of reconstituted (1:10 dilution with H6-BSA) Low-Tox® guinea pig complement (CL4051, Cedarlane, Canada) supplemented with 4 μl propidium iodide (1 mg/ml, P4170, Sigma, UK) for 15 min at 37 °C. After washing three times with H6-BSA, embryos were fixed in ice-cold ethanol supplemented with 1% Bisbenzimide H 33258 (2.5 mg/ml, B2883, Sigma, UK) at 4 °C for 1 h. For cell counting, blastocysts were washed in ice-cold fresh ethanol and mounted onto a glass microscope slide in a ~ 4 μl drop of glycerol (G5516, Sigma, UK) and coverslipped. Digital photographs of blastocysts were obtained with an inverted epifluorescence microscope (Axiovert 200 M, Carl Zeiss Ltd.) in a darkened room. Cell nuclei were manually counted with the MetaMorph software (Version 6.2r6, Molecular Devices).

### 2.5 Embryo transfer

Embryo transfer was performed by flank laparotomy in pseudopregnant MF1 recipients (7–8.5 weeks) obtained by mating with vasectomized MF1 males. On the day of ET, embryo recipients at E2.5 were anaesthetized by a single intraperitoneal injection of Ketamine (50 mg/kg, Ketaset, Pfizer, UK) and Xylazine (10 mg/kg, Rompun, Bayer, UK). Once the mouse became unresponsive to paw pinch (i.e. pedal withdrawal reflex), eyes were covered with ophthalmic ointment (Lacri-Lube, Allergan, Ireland) to prevent them from drying and the flanks were shaved and cleaned with 70% alcohol. In vitro-derived blastocysts were then washed three times with M2 medium (M7167, Sigma, UK) and loaded into a flame-polished glass pipette with a narrow opening. Immediately after, a flank incision through skin and peritoneum was made of approximately 1 cm in length, and the ovary-attached fat pad was grasped and pulled out to exteriorize the uterine horn. The uterine horn was punctured near the utero-tubal junction with a 26-gauge needle and after removal of the needle, the fire-polished glass pipette was inserted into the resultant hole and seven embryos were transferred into the uterine horn cavity with a minimal amount of medium. The reproductive tract was then gently placed back into the abdominal cavity, the peritoneum sutured, and the skin closed with wound clips. The same procedure was repeated in the opposite flank were another seven blastocysts were transferred. Following ET, recipient females were placed individually in clean cages in a warm room (28–30 °C) to recover from anaesthesia. Once recovered, females were moved to a quiet room during pregnancy and lactation. Vasectomized MF1 males were produced by exteriorizing the testes via a midline ventral incision, followed by isolation and cauterization of the vasa deferentia. The testes were then returned to the abdominal cavity and the peritoneum and skin sutured. The same pre- and post-surgery conditions used in ET were applied during vasectomy. Three weeks after surgery males were tested with two females to confirm the success of the vasectomy.

### 2.6 Offspring analysis

In the morning of the expected delivery day (i.e. 20 days pregnancy length) pups were sexed and birthweight taken. Twenty-one days after birth, offspring were marked by ear punching and weaned, allocated according to sex in groups of two to four, and weighed weekly until 27 weeks of age. Systolic blood pressure was measured at postnatal weeks 9, 15 and 21 by tail-cuff plethysmography with the Non-Invasive Blood Pressure Monitor (NIBP-8, Columbus Instruments, Columbus, Ohio, USA) [5], [21]. A training period of 2 weeks before the actual measurements was carried out to accustom the animals to the procedure. Mice were warmed before measuring blood pressure in a warm room (28–30 °C). Five readings with good waveforms and good overall quality were obtained per mouse. At 27 weeks of age a glucose tolerance test (GTT) was performed in unrestrained conscious mice after a 15-h overnight fast [21]. Glucose was measured with a blood glucose meter (Accu-Chek, Aviva, Roche Diagnostics GmbH, Germany) in small drops of blood collected by tail tipping. Topical anaesthetic cream (Lidocaine 5%, Teva, UK) was applied to the tail 20 min before starting the procedure. Following recording of the baseline glucose level (0 min), a glucose (G8270, Sigma, UK) solution (20%, in sterile, distilled water) was applied by intraperitoneal injection at a dose of 2 g/kg. Glucose levels were measured 15, 30, 60, and 120 min after glucose administration. Mice had water ad libitum during fasting and the GTT procedure. Immediately after GTT, mice were placed in clean cages with food and water ad libitum. Two days after GTT mice were culled by cervical dislocation and organs (i.e. spleen, liver, left and right kidneys, heart, and lungs) were dissected out and weighed.

### 2.7 Statistics

Statistical analysis was performed with the IBM SPSS Statistics software, version 21 (IBM Corporation). Embryo production expressed as percentage and pregnancy outcome following ET were analysed with logistic regression. The binomial test was used to examine differences in litter sex ratios. Embryo cell allocation variables and some ET outcome variables were analysed with one-way analysis of variance (ANOVA). If data did not meet the homogeneity of variance assumption (i.e. Levene's test for homogeneity of variance, P < 0.05), a Welch ANOVA was carried out. Postnatal data were converted to *Z*-scores before being analysed with a multilevel random effects regression model to compare treatment groups [5] and to analyse relationships between different readouts within each treatment group. The hierarchical structure of the data due to the mother effect was taken into consideration in the models alongside associated factors such as litter size where appropriate. The developed syntax first conducts analyses in which sex is considered a fixed effect. Since nearly all our postnatal data were affected significantly by sex, a second syntax was applied in which data were analysed separately by sex, as reported here. Of the five blood pressure readings taken per mouse, the lowest and highest values were discarded and the mean of the three middle values was used for statistical analysis [21], [22]. Area under the curve values were calculated for GTT data by the trapezoidal rule [23]. Data are presented as mean ± S.E.M. unless otherwise indicated.

## 3 Results

### 3.1 Effect of depletion of insulin and/or BCAA during embryo culture on blastocyst development and cell numbers

**Fig. 2:**
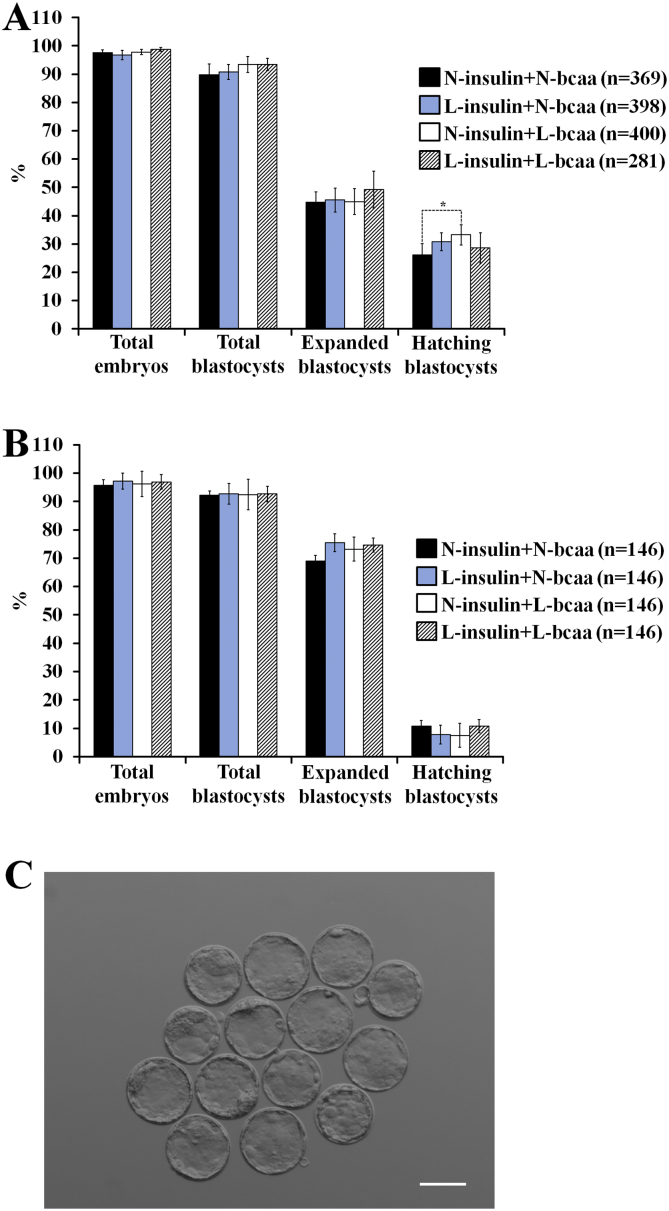
Effect of branched-chain amino acids (bcaa) and/or insulin depletion on in vitro preimplantation development in mice (Logistic regression). (**A**) Blastocyst formation (n = number of 2-cell embryos cultured) of embryos used for ET (11–16 replicates, 2–10 females per replicate). (**B**) Blastocyst formation (n = number of 2-cell embryos cultured) of embryos used for cell allocation analysis (14 replicates, 2–4 females per replicate). (**C**) Representative in vitro-derived blastocysts from the control group (N-insulin + N-bcaa) (scale bar = 100 μm). 2-cell embryos were in vitro cultured up to the blastocyst stage in either 100% (N = Normal) or 50% (L = Low) serum insulin levels with either 100% or 50% of bcaa concentrations found in the uterine luminal fluid of well-fed mice.

**Fig. 3:**
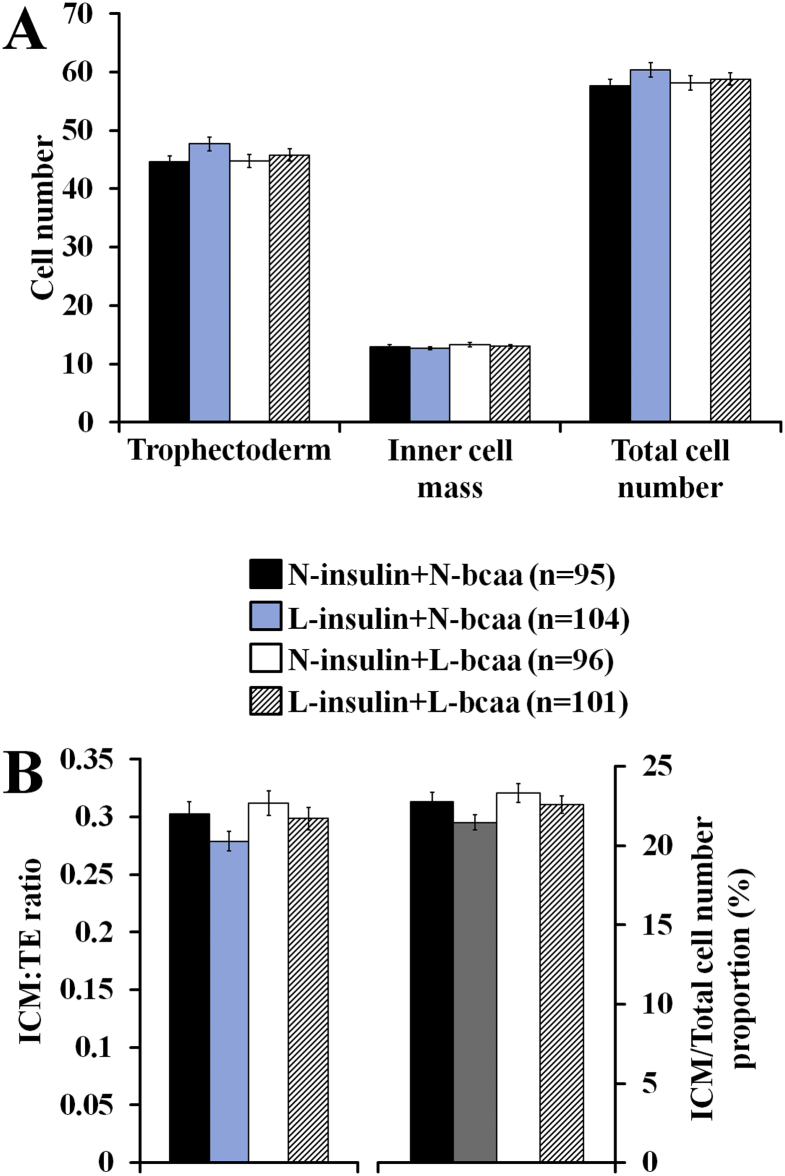
Branched-chain amino acids (bcaa) and/or insulin depletion did not affect cell number (**A**) or cell allocation variables (**B**) of expanded blastocysts in mice (n = number of blastocyst analysed) 0.14 replicates (2–4 females per replicate) (ANOVA). 2-cell embryos were in vitro cultured up to the blastocyst stage in either 100% (N = Normal) or 50% (L = Low) serum insulin levels with either 100% or 50% of bcaa concentrations found in the uterine luminal fluid of well-fed mice.

Reductions in insulin and/or BCAA did not affect the likelihood of 2-cell embryos to achieve the blastocyst stage (Fig. 2A,B,C) and the resultant blastocysts displayed no significant alterations in cell numbers within trophectoderm or ICM lineages (Fig. 3A,B). However, in embryos used for ET, 2-cell embryos exposed to N-insulin + L-bcaa had 1.4 times higher odds (95%CI = 1.02 to 1.9, P = 0.040) to achieve the hatching state when compared to the control group (Fig. 2A).

### 3.2 Effect of depletion of insulin and/or BCAA during embryo culture on pregnancy after transfer

Reductions in insulin and/or BCAA did not affect the likelihood of the embryo to achieve pregnancy or development to term. Thus, ET pregnancy rate and efficiency, litter size at term and after weaning, and offspring gender ratio were unaffected by treatment (Table 2). This is in agreement with the in vivo model of protein restriction (Emb-LPD) where no significant differences in pregnancy rates and litter size were found [5].

**Table 2:** Embryo transfer (ET) outcome of in vitro-derived embryos subjected to different concentrations of insulin and branched-chain amino acids (bcaa) exclusively during preimplantation embryo development.

Group^a^	ET pregnancy rate^b^ (%)	ET efficiency^c^ (%)	Litter size-birth^d^ (No.) [Litter No.]	Dead pups^e^ (No.)	Litter size-weaning^f^ (No.) [Litter No.]	Offspring Number	Male/Female proportion^g^
N-Insulin + N-bcaa	63.2 (12/19)	24.34 ± 5.69	5.41 ± 0.81 [12]	0.50 ± 0.23	6.55 ± 0.68 [9]	58	43.79 ± 5.99
L-Insulin + N-bcaa	66.7 (12/18)	23.85 ± 5.83	5.08 ± 0.87 [12]	1.16 ± 0.47	5.87 ± 1.23 [8]	47	56.01 ± 12.89
N-Insulin + L-bcaa	61.1 (11/18)	24.90 ± 5.17	5.72 ± 0.42 [11]	1.45 ± 0.60	5.87 ± 0.58 [8]	47	36.16 ± 7.42
L-Insulin + L-bcaa	76.9 (10/13)	30.30 ± 6.04	5.50 ± 0.68 [10]	0.10 ± 0.10	6.00 ± 0.52 [9]	54	55.37 ± 5.57

Data were analysed with ANOVA (mean ± S.E.M) except where otherwise indicated.

a Two-cell embryos were in vitro culture in 100% (normal, N) or 50% (low, L) of insulin blood levels and bcaa uterine luminal fluid concentrations found in well-fed mice.

b Dams that gave birth/total number of ET sessions performed. Analysed by logistic regression.

c Pups at birth (dead and alive)/total embryos transferred. Analysed by logistic regression.

d Calculated on dams that gave birth.

e Born dead or killed by dam before weaning.

f Calculated on dams with live pups at weaning.

g Analysed by binomial test.

### 3.3 Effect of depletion of insulin and/or BCAA during embryo culture on postnatal offspring growth and health

**Fig. 4:**
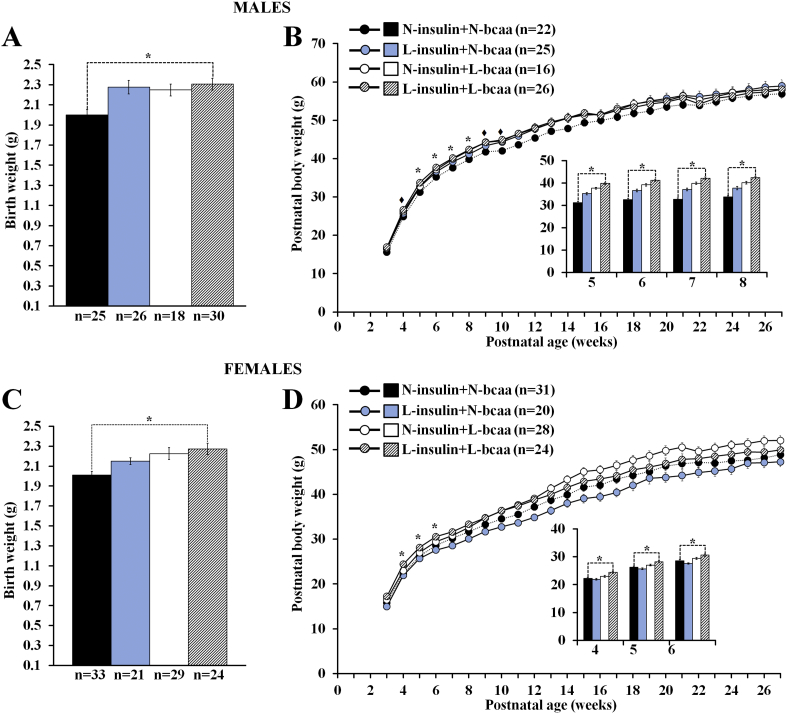
A combined depletion of insulin and branched-chain amino acids (bcaa) during in vitro preimplantation embryo development results in the production of offspring with a non sex-specific increase in birth weight (A,C) and early postnatal growth (B,D). Murine offspring is the result of transferring blastocysts (into well-fed embryo recipients) derived from 2-cell embryos cultured in either 100% (N = Normal) or 50% (L = Low) serum insulin levels with either 100% or 50% of bcaa concentrations found in the uterine luminal fluid of well-fed mice. Compared to controls (N-insulin + N-bcaa), the L-insulin + L-bcaa group showed increased birth weight and higher body weight at weeks 5–8 and 4–6 in males and females respectively. n = offspring number derived from 8 to 9 litters. Multilevel random effect regression analysis. *P < 0.05, ♦P < 0.10 (Trend).

Compared to the control group, a non-sex specific increase in birth weight was observed in pups from the groups exposed to nutrient depletion. However, this difference reached statistical significance only in offspring exposed to low concentrations of both insulin and BCAA (i.e. L-insulin + L-bcaa) (Fig. 4A,C). After weaning, male offspring from the L-insulin + L-bcaa group showed a higher body weight than their control counterparts from weeks 5 to 8, but the difference lost significance afterwards (Fig. 4B). Similarly, postnatal growth in L-insulin + L-bcaa females was enhanced in comparison to controls only from weeks 4 to 6 (Fig. 4D). Weekly weight from the other two nutrient-restricted groups (i.e. L-insulin + N-bcaa and N-insulin + L-bcaa) did not differ statistically from the control group (Fig. 4B,D).

**Fig. 5:**
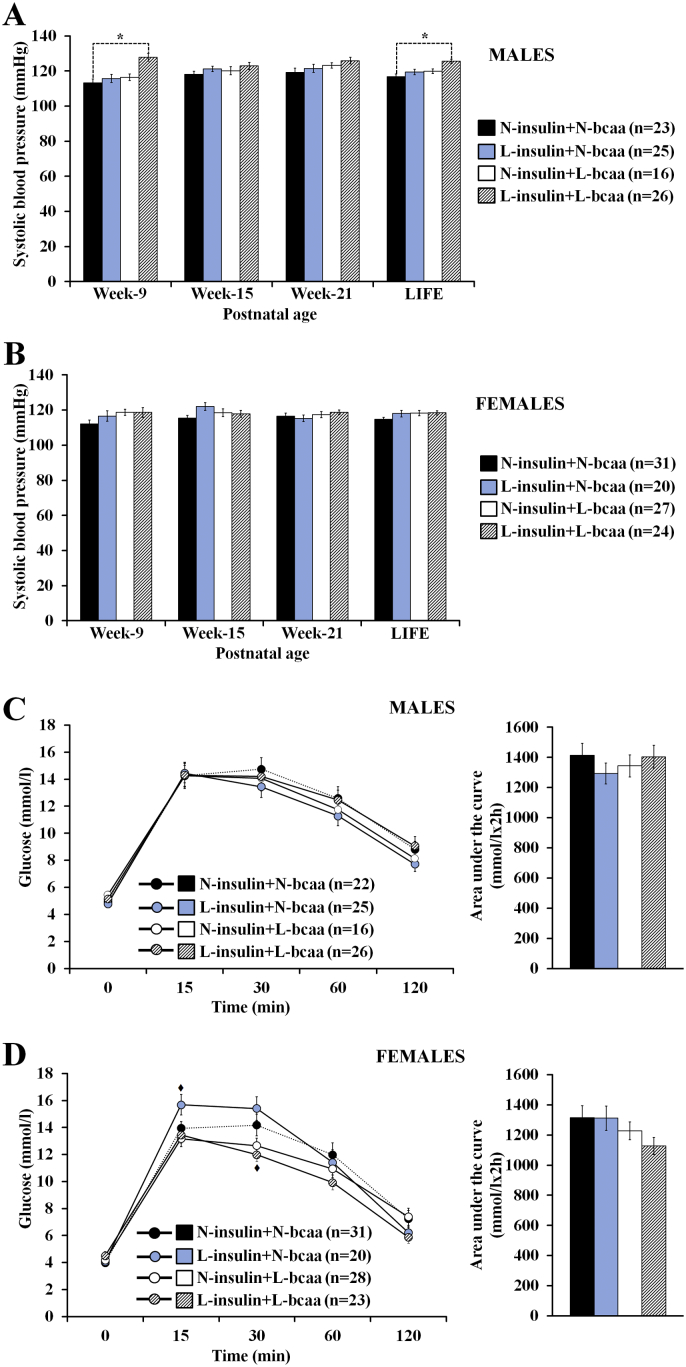
A combined depletion of insulin and branched-chain amino acids (bcaa) during in vitro preimplantation embryo development results in the production of offspring with a sex specific increase in blood pressure but with no significant changes in glucose metabolism. Murine offspring is the result of transferring blastocysts (into well-fed embryo recipients) derived from 2-cell embryos cultured in either 100% (N = Normal) or 50% (L = Low) serum insulin levels with either 100% or 50% of bcaa concentrations found in the uterine luminal fluid of well-fed mice. “LIFE” indicates the mean value of the measurements done during postnatal life (i.e. at weeks 9, 15 and 21). Compared to controls (N-insulin + N-bcaa), L-insulin + L-bcaa males showed increased systolic blood pressure at week 9. n = offspring number derived from 8 to 9 litters. Multilevel random effect regression analysis. *P < 0.05, ♦P < 0.10 (Trend).

Systolic blood pressure was measured non-invasively at postnatal weeks 9, 15, and 21 and the mean value for these times recorded as ‘LIFE’ (Fig. 5A,B). In both male and female offspring the LIFE value was higher in all three treatment groups compared with the control. However, only in male offspring, and specifically the L-insulin + L-bcaa group, was a significantly increased systolic blood pressure recorded both at week 9 and LIFE compared with the control group (Fig. 5A). The subsequent readings of the L-insulin + L-bcaa male group remained higher than the control group but it did not reach statistical significance. Systolic blood pressure was not significantly affected in female offspring after embryo nutrient depletion (Fig. 5B).

An intraperitoneal glucose tolerance test was carried out at postnatal week 27. Glucose levels at different time points after glucose administration were not significantly affected in male and female offspring (Fig. 5C,D). Only a tendency value was observed among the female groups. As such, compared to the control group, females in the L-insulin + N-bcaa group have a greater peak of glucose concentration 15 min after glucose injection (P = 0.056), whereas females in the L-insulin + L-bcaa showed a lower peak of glucose concentration 30 min after glucose treatment (P = 0.080). Area under the curve was not affected in both sexes (Fig. 5C,D).

**Fig. 6:**
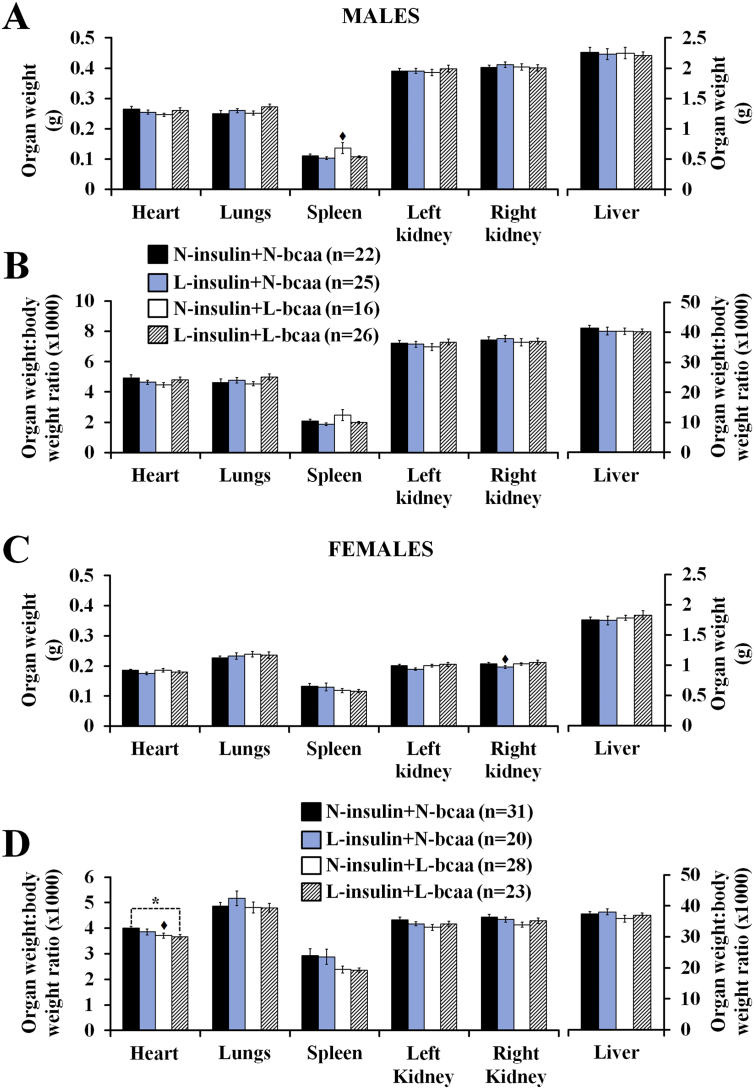
A combined depletion of insulin and branched-chain amino acids (bcaa) during in vitro preimplantation embryo development results in the production of offspring with no significant changes in organ weight, but slight alteration in organ allometry. Murine offspring is the result of transfer of blastocysts (into well-fed embryo recipients) derived from 2-cell embryos cultured in either 100% (N = Normal) or 50% (L = Low) serum insulin levels with either 100% or 50% of bcaa concentrations found in the uterine luminal fluid of well-fed mice. Compared to controls (N-insulin + N-bcaa), L-insulin + L-bcaa females showed increased heart:body weight ratio. n = offspring number derived from 8 to 9 litters. Multilevel random effect regression analysis. *P < 0.05, ♦P < 0.10 (Trend).

Organ weight analysis at the end of the study at postnatal week 27 did not reveal significant changes in male and female offspring (Fig. 6A,C). Only tendency values were observed, where, compared to the control group, males in the N-insulin + L-bcaa group had a heavier spleen (P = 0.081) and females in the L-insulin + N-bcaa group had a lighter right kidney (P = 0.052). Nevertheless, compared to the control group, females in the L-insulin + L-bcaa group displayed a low heart weight to body weight ratio (Fig. 6D). A similar trend was observed in the N-insulin + L-bcaa female group, but the difference did not reach statistical significance (P = 0.068). Organ weight:body weight ratios were not affected by treatment in male offspring (Fig. 6B).

The relationship between different postnatal outcomes within each treatment group was examined and key differences found between treatment groups are shown in Table 3. Weaning weight positively correlated with systolic blood pressure both at week 9 and the LIFE score in control male and female offspring (P < 0.05), however, these associations were lost in both the L-insulin + L-bcaa and N-insulin + L-bcaa male and female offspring but broadly retained in the L-insulin + N-bcaa male and female offspring (Table 3). No relationship was evident in controls between body weight and glucose tolerance AUC except for weight in males at week 27 when the GTT was conducted; a similar lack of relationship was found in the L-insulin + N-bcaa and N-insulin + L-bcaa groups except in the latter for female weight at week 9. In contrast, in both male and especially female L-insulin + L-bcaa offspring, body weight at different ages was commonly positively correlated with glucose tolerance AUC at 27 weeks of age (P < 0.05–0.001; Table 3). Lastly, control and the single nutrient depletion groups showed little relationship at different ages between fasted, peak (15 min) or end (120 min) glucose levels measured during the GTT at 27 weeks of age. In the L-insulin + L-bcaa offspring, however, extensive positive correlations existed between body weight at all ages and glucose levels at 27 weeks, especially in the females (P < 0.05–0.001; Table 3). Thus, there is evidence that in L-insulin + L-bcaa offspring there is disturbance in normal weight-physiology relationships including associations between early and later postnatal characteristics.

**Table 3:** Phenotypic correlations of offspring generated after embryo transfer of in vitro-derived embryos subjected to different concentrations of insulin and branched-chain amino acids (bcaa) exclusively during preimplantation embryo development.

	N-insulin + N-bcaa		L-insulin + N-bcaa		N-insulin + L-bcaa		L-insulin + L-bcaa	
	Male [22]	Female [31]	Male [24]	Female [21]	Male [16]	Female [27]	Male [26]	Female [23]
**Blood pressure and weight at 3 weeks of age**								
SBP wk9 – weaning weight	0.626⁎	0.473⁎	0.392$	0.721⁎⁎	0.124	− 0.193	0.312	0.026
SBP wk15 – weaning weight	0.417	0.134	0.433$	0.121	0.465$	− 0.221	0.215	0.210
SBP wk21 – weaning weight	0.514$	0.261	0.302	0.243	− 0.389	0.195	− 0.038	0.160
SBP LIFE – weaning weight	0.746⁎	0.437⁎	0.503⁎	0.425$	0.145	− 0.106	0.312	0.185

**Glucose tolerance at 27 weeks and weight at different ages**								
AUC – bw wk3	0.076	− 0.086	− 0.276	0.101	− 0.383	− 0.195	0.337	0.379$
AUC – bw wk9	0.065	0.025	− 0.281	0.254	− 0.428	− 0.470⁎	0.072	0.546⁎
AUC – bw wk15	0.202	− 0.05	− 0.113	0.137	− 0.344	− 0.302	0.372$	0.663⁎
AUC – bw wk21	0.352	0.079	− 0.062	0.170	0.122	− 0.185	0.512⁎	0.660⁎⁎
AUC – bw fasted	0.427$	0.255	0.015	0.118	0.264	− 0.067	0.597⁎	0.688⁎⁎

**GTT dynamics at 27 weeks and weight at different ages**								
Fasted glucose – bw3	0.103	0.101	− 0.415⁎	− 0.029	− 0.288	0.105	0.335	0.645⁎
GTT 15 min – bw3	0.075	0.034	− 0.097	− 0.200	− 0.152	− 0.217	0.401	0.328
GTT 120 min – bw3	− 0.035	− 0.209	− 0.082	− 0.010	− 0.346	− 0.103	0.362	0.563⁎
Fasted glucose – bw9	− 0.079	− 0.041	− 0.067	− 0.025	0.011	0.073	− 0.020	0.765⁎
GTT 15 min – bw9	0.089	− 0.026	− 0.009	0.134	− 0.211	− 0.299	0.005	0.527⁎
GTT 120 min – bw9	0.091	0.132	− 0.180	0.504⁎	− 0.283	− 0.198	0.107	0.604⁎
Fasted glucose – bw15	0.005	− 0.075	0.009	− 0.027	− 0.039	0.252	0.106	0.617⁎⁎
GTT 15 min – bw15	0.131	− 0.042	0.105	0.064	− 0.148	− 0.193	0.214	0.613⁎⁎
GTT 120 min – bw 15	0.271	0.034	− 0.079	0.301	0.072	0.044	0.404$	0.679⁎⁎
Fasted glucose – bw21	0.068	− 0.265	− 0.063	0.103	− 0.100	0.320$	0.226	0.620⁎⁎
GTT 15 min – bw21	0.105	0.038	0.132	0.073	0.103	− 0.124	0.374$	0.618⁎⁎
GTT 120 min – bw21	0.421$	0.177	− 0.042	0.327	0.401	0.093	0.530⁎	0.689⁎⁎
Fasted glucose – bw fasted	0.220	− 0.274	− 0.033	0.171	− 0.021	0.379⁎	0.395⁎	0.626⁎⁎
GTT 15 min – bw fasted	0.119	0.221	0.130	0.065	0.200	− 0.020	0.531⁎	0.631⁎⁎
GTT 120 min – bw fasted	0.514⁎	0.262	0.039	0.216	0.434$	0.127	0.498⁎	0.650⁎⁎

Data were analysed after z-standardisation using random effects regression within each treatment group split by sex. Correlation coefficients are shown and statistically relevant relationships are marked. bw: body weight; [ ] offspring number.

$ P < 0.1.

⁎ P < 0.05.

⁎⁎ P < 0.001.

## 4 Discussion

The main objective of our study was to determine whether insulin and/or BCAA depletion during early development of the embryo can act as inductive factors of altered phenotypes during postnatal life. This was primarily to test the hypothesis that depletion in these metabolites following maternal Emb-LPD during blastocyst formation was causative in the subsequent postnatal phenotype of offspring from the dietary model [24]. The current IVEC-ET model, using the same MF1 mouse strain as used in the Emb-LPD model, attempted in the control group (N-insulin + N-bcaa) to mimic the embryo compositional environment for both AAs detected in ULF and the physiological level of serum insulin found in control diet mothers. This culture medium is quite distinct in composition to that found in KSOM + AA with multi-fold differences evident in the levels of many AAs [8], [19] hence novel for mouse embryo culture studies yet more closely resembling the in vivo conditions experienced by preimplantation embryos within the uterus.

The three experimental culture treatments only differed with respect to insulin and/or combined BCAA concentrations which at 50% reduction mimicked the conditions found in the Emb-LPD exposed embryo environment [8]. Our reasoning that these metabolites during preimplantation development may be the inductive factors in dietary programming were that (i) Emb-LPD blastocysts show significant reduction in the growth-regulating mTORC1 signalling which is mediated through insulin and BCAA sensing [8]; (ii) Emb-LPD blastocysts are already programmed for longer-term changes in phenotype as evidenced by ET to normal fed recipients [5]. Moreover, there is also circumstantial evidence in the literature that embryos are equipped with insulin receptors and AA transporters and that exposure to these metabolites cause changes in later fetal growth rate (discussed later). Lastly, the notion that growth rate regulators are prime candidates for preimplantation programming inductive factors is supported by the observation that perinatal weight in offspring from Emb-LPD and LPD (where the diet is maintained for whole of gestation) treatments positively correlates with later adult weight and disease severity [5].

The main outcomes from the use of the IVEC-ET model were that blastocyst development and lineage-specific cell number were not affected by the depleted metabolites but that aspects of postnatal phenotype were, including increased birth weight and early growth rate in both genders; increased systolic blood pressure in male offspring in early life; no clear effect on glucose tolerance; reduced heart body weight ratio in female offspring; and disturbance in weight-physiology relationships. Such effects are generally most prevalent in the L-insulin + L-bcaa group. When compared to the Emb-LPD phenotype there are some similarities but also distinctions. Thus, blastocyst development rate is unaffected by Emb-LPD but trophectoderm and total cell numbers are increased [8]. Postnatally, birth weight is increased by Emb-LPD and for females, this growth advantage is maintained throughout life; systolic blood pressure is increased in both genders; and heart/body weight ratio is reduced in female offspring [5]. Glucose tolerance in Emb-LPD offspring was not measured as in the current study but glucose and insulin serum levels were normal in both genders [7]. Thus, the IVEC-ET model replicates well the Emb-LPD model in terms of postnatal characteristics (birth weight, early growth, blood pressure, heart sizing) but generally these outcomes may be less pronounced and gender-relatedness may not be consistent. However, the blastocyst phenotype of Emb-LPD is not replicated in the current study.

Our study is therefore the first to show that direct exposure to low levels of both insulin and BCAA exclusively during preimplantation period is sufficient to induce an increment in both body weight gain and blood pressure during early postnatal life. It also gives support to our hypothesis that insulin and BCAA are important nutritional mediators involved in the programming of postnatal disease by protein undernutrition at the early embryo stage [4]. Our study further confirms in general terms the critical relevance of the mammalian preimplantation period as a developmental window where programming with long-term consequences for health and disease risk can be exerted and evident in both rodent and large animal species [5], [21], [22], [25], [26], [27], [28], [29], [30], [31], [32], [33], [34].

In a broader perspective, this periconceptional window where embryo culture conditions may affect postnatal phenotype is also pertinent to the human and assisted reproduction treatment (ART). Although controversy exist on the role of the culture medium as determinant of birth weight in human IVF [35], [36], [37], [38], our experimental work here and a new randomised control trial in the human [39], strongly indicate that the composition of the culture medium is an important contributing factor for birth weight variation in assisted reproduction. ART children further display increased risk of cardiovascular dysfunction likely to derive to their in vitro treatments rather than factors associated with parental infertility, including relative hypertension [40], [41], [42] together with cardiovascular remodeling during pregnancy resulting in altered heart shape and chamber size [41], [43]. Thus, across small and large animal models, and in human ART, embryo culture conditions and composition are critical factors determining postnatal phenotype – yet, in clinical practice, commercial culture media composition is undisclosed [38].

We consider first the short-term outcomes of our treatments that had no effect on blastocyst phenotype, in contrast to the Emb-LPD blastocyst phenotype. Studies in mice have shown that insulin and AA, including BCAA, added to culture medium can exert stimulatory effects. For instance, AA supplementation can promote blastocyst formation, cell proliferation [44], [45], [46], [47], glucose uptake [14] and outgrowth formation [11]. Similarly, supplementation with insulin can increase the formation [15] and cell number of blastocysts [48], [49] along with reduced protein degradation [50], increased protein synthesis [51], [52], [53] and enhanced endocytosis with resultant increased protein intake [50], [54]. These positive effects of insulin and AA supplementation act through the presence of preimplantation insulin receptors [51], [55], [56] and amino acid transporters (AAT) including system L and system b0,+ AATs responsible for BCAA transport [57], [58], [59]. Furthermore, it has been suggested that insulin can act synergistically with AA to improve embryo development in vitro [60].

The absence of effect of low insulin and/or BCAA on blastocyst phenotype in the current study suggests that these factors are not inductive for the increased trophectoderm proliferation seen in the Emb-LPD model. This is supported by the evidence that (i) non-essential AAs rather than BCAAs may have a predominant effect on mouse embryo proliferation in vitro [45] and that (ii) for insulin, whilst the concentrations used here match those used previously, the stimulatory effect has been on ICM rather than trophectoderm cell numbers [49]. Moreover, the effect of maternal Emb-LPD on increasing trophectoderm proliferation in the mouse is not matched in the rat where trophectoderm cell numbers decrease [61], suggesting it is not a conserved response across species. This view further suggests that different features of the compensatory response in extra-embryonic lineages activated by Emb-LPD treatment (see Introduction, second paragraph) may be separately regulated. Thus, in an earlier study, we found that the stimulation of mouse blastocyst trophectoderm endocytosis as occurring after Emb-LPD could be induced in vitro through low BCAA concentration (equivalent to that used here) in the presence of normalised insulin [9]. Alternative maternal environmental factors changing in response to Emb-LPD treatment should therefore be assessed in vitro for evidence of their role in the early programming events in this model; for example, this could include oestrogen, glucose or other AAs or their combination with insulin [8], or other factors found to alter in response to maternal undernutrition in other models [62].

In contrast to the preimplantation blastocyst phenotype, we have found that embryo culture in depleted insulin and BCAA replicates well the postnatal phenotype programmed by Emb-LPD. How might these long-term outcomes be so induced by periconceptional metabolite levels? Increased fetus and birth weight have been reported in mice derived from the transfer of blastocysts that were in vitro-exposed to insulin from the 2-cell stage [15], [16]. Embryo culture with amino acids is also a positive factor in fetal development [14]. However, in the current study, increased gestational growth occurs when preimplantation metabolites are *depleted*. Our Emb-LPD studies show that the low insulin and BCAA maternal environment associates with altered blastocyst mTORC1 signalling and compensatory extra-embryonic responses that collectively enhance maternal nutrient supply and lead to increased fetal-placental efficiency [5], [8], [10]. Moreover, these extra-embryonic responses include epigenetic modifications that can be traced back to the preimplantation period [63]. Thus, we propose that preimplantation insulin and BCAA depletion, through activation of extra-embryonic responses, can lead to increased gestational growth and birth weight as shown here. Moreover, in the Emb-LPD model, after release from the dietary challenge, the embryonic somatic lineages activate a separate mechanism to stimulate ribosome biogenesis through increased rRNA expression, also epigenetically regulated [64]. This latter mechanism provides the opportunity to maximise the benefit of extra-embryonic adaptations and forms the basis for ‘catch-up’ growth to occur, a common feature in many developmental programming models [2]. The resulting increased birth weight and early postnatal growth rate may then directly associate with increased chronic disease risk in adulthood as found in the Emb-LPD model [5]. The molecular and signalling pathways linking preimplantation insulin and BCAA levels with the increased fetal growth trajectory after Emb-LPD release are yet to be defined but convergence through the mTORC1 pathway is a prime candidate. Thus, the ribosome factor identified in stimulating ribosome biogenesis through reducing rDNA methylation after release from maternal Emb-LPD, Rrn3 (TIF-1A), is sensitive to nutrient levels and mTORC1 signalling [65], [66]. It is also worth highlighting that, similar to the Emb-LPD model [5], after depletion of BCAA and insulin preimplantation, early age body weight is a strong predictor of metabolic health measured several weeks later, especially in female offspring. In contrast, offspring from single or no nutrient depletion preimplantation show no or weak relationships between body weight at any age and glucose handling capability (Table 3). This suggests that depletion of two key nutrients preimplantation may induce a tight co-regulation of metabolism and body weight very early on in life. Underlying mechanisms warrant further investigation.

It is relevant to highlight that our postnatal data were subjected to a robust statistical analysis in which factors such as the random mother effect (i.e. embryo recipient) and gestational litter size were included where appropriate and considering interpretation complications through collinearity. However, litter size can be critical for the proper analysis of in utero programming data from litter-bearing animals [67] and often is ignored in the statistical analyses of DOHaD-related studies working with polyovulatory species, which can result in type 1 errors. Here it is also important to consider some factors that could have influenced our results. Our in vitro embryo culture was carried out under atmospheric O_2_ conditions (~ 20%), and it is known that a low O_2_ environment (i.e. 5%) promotes a better utilization of AA in mouse preimplantation embryos [68]. Also, unlike microfluidic systems where a constant flow of medium can be regulated [69], in our static in vitro system consumption and degradation of culture medium components took place without renewal, hindering a constant exposure of nutrients tested in our study. Although it remains to be determined if these refinements (i.e. low O_2_ and microfluidics) could exacerbate the postnatal phenotype observed in our study, we believe our data come from a robust in vitro embryo culture model.

In conclusion, our study has demonstrated that an in vitro model to recreate the maternal metabolite conditions of low insulin and BCAA levels generated through maternal dietary protein restriction (Emb-LPD) are sufficient in embryo culture to induce the postnatal growth and chronic disease characteristics previously reported for this dietary treatment. Our work further highlights the criticality of preimplantation environment and nutrition for long-term health and disease risk and has clear relevance to ART practice. A future goal for clinical benefit will be to expand our understanding of the molecular, epigenetic and signalling pathways that link preimplantation exposure with adult phenotype and metabolism.

## Transparency document

Transparency document. Image 1
